# Effectiveness of using a meditation app in reducing anxiety and improving well-being during the COVID-19 pandemic: A structured summary of a study protocol for a randomized controlled trial

**DOI:** 10.1186/s13063-020-04935-6

**Published:** 2020-12-09

**Authors:** Katherine T. O’Donnell, Melanie Dunbar, Diana L. Speelman

**Affiliations:** Lake Erie College of Osteopathic Medicine, Erie, PA USA

**Keywords:** COVID-19, Randomized controlled trial, protocol, mindfulness, well-being, anxiety, meditation app

## Abstract

**Objectives:**

This interventional study will investigate the effect of daily use of a mindfulness app on measures of participant anxiety, well-being, and perceived outlook during the COVID-19 pandemic, by comparing pre-intervention survey responses to post-intervention survey responses.

**Trial design:**

Randomized, controlled trial with parallel assignment. Adults will be assigned either to daily use of a meditation app for 30 days or to a control group (no usage of meditation app) with a 1:1 equivalence allocation ratio.

**Participants:**

Inclusion Criteria: Participants must be 18 or older, have a smartphone, able to download apps to their smartphone, must be fluent in the English language, able to complete surveys on their own, and must be in the United States for the duration of the study. Exclusion Criteria: Current regular use of a mindfulness or meditation app, regular practice of mindfulness or meditation, regular therapy sessions, inability to complete surveys independently, or any mental health restrictions that would prevent participation. All data will be collected through the Insight Timer Meditation App and Google Forms. This trial is being conducted through the Lake Erie College of Osteopathic Medicine in Erie, PA, with all data collected digitally.

**Intervention and comparator:**

Intervention: Participants will be sent a link to a pre-intervention survey prior to first use of the mindfulness app. Participants will be instructed to use the Insight Timer app for 10 minutes daily for 30 days. At the end of the 30-day intervention period, participants will be sent a link for the post-intervention survey. Two months after the conclusion of the 30-day intervention period, participants will be sent a link for another post-intervention survey. Comparator: Participants will receive the same surveys, but will not use any mindfulness app for the 30-day intervention period. After this 30-day period, participants are invited to use the Insight Timer app if they so choose.

**Main outcomes:**

The main outcomes are (1) anxiety as assessed by survey questions adapted from the GAD7, comparing pre-intervention to post-30-days of app usage and (2) well-being as assessed by survey questions adapted from the WHO-5, comparing pre-intervention and post-30-days of app usage.

**Randomization:**

Participants will be allocated to interventions via a block random sequence generator with a 1:1 allocation ratio in blocks of 8.

**Blinding (masking):**

No masking is being used in this study (open label).

**Numbers to be randomized (sample size):**

Approximately 75 participants will be randomized to each group, with an estimated enrollment of 150 participants.

**Trial status:**

This study is protocol version number 27-126 and was approved on May 10, 2020. Recruitment began on August 19, 2020 and will end February 28, 2021. The study is estimated to complete on April 30, 2021.

**Trial registration:**

This trial was registered to ClinicalTrials.gov on 30 April 2020. The ClinicalTrials.gov Identifier is NCT04369378.

**Full protocol:**

The full protocol is attached as an additional file, accessible from the Trials website (Additional File 1). In the interest in expediting dissemination of this material, the familiar formatting has been eliminated; this Letter serves as a summary of the key elements of the full protocol.

## Supplementary Information

**Additional file 1.** Full Study Protocol.

## Data Availability

De-identified participant data will be reported as individual data points, as well as means +/- SD or medians with quartiles upon publication of findings. Access criteria to the final trial dataset will be upon written request to the corresponding author on publication.

