# Supplementary material for: Effectiveness of using a meditation app in reducing anxiety and improving well-being during the COVID-19 pandemic: A structured summary of a study protocol for a randomized controlled trial
Source: Trials. 2020 Dec 9;21:1006. doi: 10.1186/s13063-020-04935-6 (PMC7724449; doi:10.1186/s13063-020-04935-6)
Supplement: Supplementary file 1 — Additional file 1. Full Study Protocol. [file 13063_2020_4935_MOESM1_ESM.pdf]

COVID-19 is an emerging, rapidly evolving situation.
Get the latest public health information from CDC: https://www.coronavirus.gov.
Get the latest research information from NIH: https://www.nih.gov/coronavirus.

Effectiveness of Using a Meditation App in Reducing Anxiety and Improving Well-being During the Covid-19 Pandemic

The safety and scientific validity of this study is the responsibility of the study sponsor and investigators. Listing a study does not mean it has been evaluated by the U.S. Federal Government. Know the risks and potential benefits of clinical studies and talk to your health care provider before participating. Read our disclaimer for details.

ClinicalTrials.gov Identifier: NCT04369378

Recruitment Status : Recruiting
First Posted : April 30, 2020
Last Update Posted : November 13, 2020

See Contacts and Locations

Sponsor:
Lake Erie College of Osteopathic Medicine

Information provided by (Responsible Party):
Diana Speelman, Ph.D., Lake Erie College of Osteopathic Medicine

Study Description

Go to

Brief Summary:
This interventional study will investigate the effect of daily use of a mindfulness app on measures of participant anxiety, well-being, and future outlook during the Covid-19 pandemic, by comparing pre-intervention survey responses to post-intervention survey responses.

Condition or disease	Intervention/treatment	Phase
Anxiety<br>Well-being	Behavioral: Meditation app usage	Not Applicable

Detailed Description:

Question 1: Can 30 days of daily use of a mindfulness app during the Covid-19 pandemic reduce anxiety and improve general well-being and future outlook? Outcomes measured: Pre- and post-intervention surveys to assess well-being and anxiety (primary outcomes), future outlook, hopefulness, and sleep habits (secondary outcomes). Many of these survey questions come from well-validated surveys (WHO-5 Well-being survey, GAD7 Anxiety survey). Post intervention surveys will be administered at the end of the 30d intervention, as well as 2 months after the completion of the 30d intervention.

Question 2: Can 30 days of daily use of a mindfulness app during the Covid-19 pandemic improve nutritional habits? Outcomes measured: Pre- and post-intervention surveys to assess general nutrition habits, including frequency of consuming whole foods (fruits, vegetables, whole grains) and frequency of consuming prepared meals (secondary outcomes). Post intervention surveys will be administered at the end of the 30d intervention, as well as 2 months after the completion of the 30d intervention.

Question 3: Will study participants be more likely to continue to use the mindfulness app after completion of the study? Outcomes measured: Post-intervention survey administered at the end of the 30d intervention will be used to assess likelihood to continue using app and frequency of anticipated continued use. The post-intervention survey administered 2 months after the completion of the 30d intervention will ask if participants continued to use the app, and the frequency of use (secondary outcomes).

Study Design

Go to

Study Type :
Interventional (Clinical Trial)

Estimated Enrollment :
200 participants

Allocation:
Randomized

Intervention Model:
Parallel Assignment

Intervention Model Description:
Randomized controlled trial: adults randomly assigned to daily use of a meditation app for 30 days or control group (no usage of meditation app).

Masking:
None (Open Label)

Primary Purpose:
Treatment

Official Title:
Effectiveness of Using a Meditation App in Reducing Anxiety and Improving Well-being During the Covid-19 Pandemic

Actual Study Start Date :
August 19, 2020

Estimated Primary Completion Date :
February 28, 2021

Estimated Study Completion Date :
April 30, 2021

Resource links provided by the National Library of Medicine

MedlinePlus related topics: Anxiety

U.S. FDA Resources

Arms and Interventions

Go to

Arm	Intervention/treatment
Experimental: Meditation app group<br>Participants will also be given access to the mindfulness app (Insight Timer), and instructed to use it for 10 min daily for 30 days. Two days before the end of the 30 day intervention period, participants will be sent a link to a Google Form	Behavioral: Meditation app usage<br>Investigators will confirm eligibility of applicants, enroll participants in the study and provide an identification number for de-identification of the data, and provide a list of mental health resources to participants. Participants will then

for the post-intervention survey and will be asked to complete the survey within the next 3 days. Two months after the conclusion of the 30 day intervention period (90 days after study began), participants will be sent a link to a Google Form for another post-intervention survey, and asked to complete it within 5 days.	be sent a link to a Google Form for the pre-intervention survey, to be completed prior to first use of the mindfulness app. Participants will also be given access to the mindfulness app (Insight Timer), and instructed to use it for 10 min daily for 30 days. Two days before the end of the 30 day intervention period, participants will be sent a link to a Google Form for the post-intervention survey and will be asked to complete the survey within the next 3 days. Two months after the conclusion of the 30 day intervention period, participants will be sent a link to a Google Form for another post-intervention survey, and asked to complete it within 5 days.
No Intervention: Control group<br>Participants will be in the no intervention period for 30 days. Two days before the end of the 30 day no intervention period, participants will be sent a link to a Google Form for the post-intervention survey and will be asked to complete the survey within the next 3 days. After this 30 day no intervention period, participants are invited to use the Insight Timer app if they so choose. Two months after the conclusion of the 30 day no intervention period (90 days after study began), participants will be sent a link to a Google Form for another post-intervention survey, and asked to complete it within 5 days.	

Outcome Measures

Go to

Primary Outcome Measures :

1. Anxiety [Time Frame: Immediate post-intervention (compare with pre-intervention data)]

Assessed by survey questions (in part adapted from GAD7)

2. Well-being [Time Frame: Immediate post-intervention (compare with pre-intervention data)]

   Assessed by survey questions (in part adapted from WHO-5)

Secondary Outcome Measures :

1. Future outlook & hopefulness [Time Frame: Immediate post-intervention (compare with pre-intervention data)]

   Assessed by survey questions

2. Sleep habits [Time Frame: Immediate post-intervention (compare with pre-intervention data)]

   Assessed by survey questions

3. Nutrition habits [Time Frame: Immediate post-intervention (compare with pre-intervention data)]

   Assessed by survey questions (frequency of consuming whole foods and prepared meals)

4. Meditation app continued usage [Time Frame: Immediate post-intervention (compare with pre-intervention data)]

   Assessed by survey questions (anticipated and actual continued usage of app)

5. Anxiety [Time Frame: 2 months post-intervention (compare with pre-intervention, immediate post-intervention data)]

   Assessed by survey questions (in part adapted from GAD7)

6. Well-being [Time Frame: 2 months post-intervention (compare with pre-intervention, immediate post-intervention data)]

   Assessed by survey questions (in part adapted from WHO-5)

7. Future outlook & hopefulness [Time Frame: 2 months post-intervention (compare with pre-intervention, immediate post-intervention data)]

   Assessed by survey questions

8. Sleep habits [Time Frame: 2 months post-intervention (compare with pre-intervention, immediate post-intervention data)]

   Assessed by survey questions

9. Nutrition habits [Time Frame: 2 months post-intervention (compare with pre-intervention, immediate post-intervention data)]

   Assessed by survey questions (frequency of consuming whole foods and prepared meals)

10. Meditation app continued usage [Time Frame: 2 months post-intervention (compare with immediate post-intervention data)]

    Assessed by survey questions (anticipated and actual continued usage of app)

Eligibility Criteria

Go to

Information from the National Library of Medicine

NIH NLM

Choosing to participate in a study is an important personal decision. Talk with your doctor and family members or friends about deciding to join a study. To learn more about this study, you or your doctor may contact the study research staff using the contacts provided below. For general information, [Learn About Clinical Studies.]

Ages Eligible for Study:
18 Years and older (Adult, Older Adult)

Sexes Eligible for Study:
All

Accepts Healthy Volunteers:
Yes

Criteria

Inclusion Criteria:

- Participants must be 18 or older. Inclusion criteria are access to a smartphone and ability to download

the mindfulness app, fluency in English, and the ability to complete surveys independently.

Exclusion Criteria:

- Exclusion criteria include current regular use of a mindfulness or meditation app, regular practice of mindfulness or meditation, regular therapy sessions, inability to complete surveys independently, or any mental health restrictions that would prevent them from participating.

Contacts and Locations

Go to

Information from the National Library of Medicine

NIH NLM

To learn more about this study, you or your doctor may contact the study research staff using the contact information provided by the sponsor.

Please refer to this study by its ClinicalTrials.gov identifier (NCT number): ***NCT04369378***

Contacts

Contact: Diana L Speelman, PhD 814-868-1113 dspeelman@lecom.edu

Contact: Melanie Dunbar, PhD 814-866-8160 mdunbar@lecom.edu

Locations

United States, Pennsylvania

LECOM **Recruiting**

Erie, Pennsylvania, United States, 16509

Contact: Diana Speelman, PhD 814-868-1113 dspeelman@lecom.edu

Sponsors and Collaborators

Lake Erie College of Osteopathic Medicine

Investigators

Principal Investigator: Diana Speelman, PhD Lake Erie College of Osteopathic Medicine

More Information

Go to

Responsible Party:

Diana Speelman, Ph.D., Director of Research, Associate Professor of Biochemistry, Lake Erie College of Osteopathic Medicine

ClinicalTrials.gov Identifier:

NCT04369378 History of ChangesHistory of Changes

Other Study ID Numbers:

27-126

First Posted:

April 30, 2020 Key Record Dates

Last Update Posted:

November 13, 2020

Last Verified:

November 2020

Individual Participant Data (IPD) Sharing Statement:

Plan to Share IPD:

Yes

Plan Description:

De-identified participant data will be reported as individual data points, as well as means +/- SD or medians with quartiles.

Supporting Materials:

Study Protocol

Statistical Analysis Plan (SAP)

Time Frame:

Upon publication of findings

Access Criteria:

Upon written request to corresponding author on publication

Studies a U.S. FDA-regulated Drug Product:

No

Studies a U.S. FDA-regulated Device Product:

No

Additional relevant MeSH terms:

- Anxiety Disorders
- Mental Disorders
